# Blood Cell Imaging Analysis for Multiple Sclerosis Classification Using Leave-One-Subject-Out Study with Deep Neural Networks

**DOI:** 10.3390/jimaging12100482

**Published:** 2026-10-03

**Authors:** Dylan Tijburg, Noor Christoph, Tahir Nawaz, Jan De Zoeten, Ian Galea, Carmen Jacob, Marya Butt

**Affiliations:** 1 Data Driven Smart Society, Inholland University of Applied Sciences, Bergerweg 200, 1817 MN Alkmaar, The Netherlands; 2 Upland Workshop, Los Angeles, CA 90049, USA; tahir@uplandworkshop.com; 3 RR Mechatronics BV, 1689 AN Zwaag, The Netherlands; 4 Clinical Neurosciences, Clinical & Experimental Sciences, Faculty of Medicine, University of Southampton, Southampton SO16 6YD, UKc.jacob@soton.ac.uk (C.J.); 5 Wessex Neurological Center, Southampton General Hospital, University Hospital Southampton NHS Foundation Trust, Southampton SO16 6YD, UK

**Keywords:** multiple sclerosis, red blood cell classification, deep learning, Swin Transformer, VGG16, transfer learning, leave-one-out cross-validation, explainable AI, Grad-CAM, medical image analysis

## Abstract

Multiple sclerosis (MS) is a chronic neurological disease whose diagnosis typically relies on resource-intensive and partly invasive procedures such as MRI and spinal fluid analysis. This study explores whether deep learning can identify distinguishing patterns in RBC images associated with MS, aiming to determine whether blood-cell imaging may provide information relevant to understanding the disease. The initial dataset comprised 121 images from 13 independent subjects. To expand the dataset to 1944 images, image augmentation techniques were applied to the training data to increase image variability. Due to the small dataset, training was carried out using a Leave-One-Subject-Out (LOSO) strategy to assess model performance on unseen subjects. Four deep learning architectures, VGG16, DenseNet201, MobileNetV2, and the Swin Transformer, were evaluated using subject-level Leave-One-Out Cross-Validation (LOOCV). The Swin Transformer achieved the highest mean accuracy (73.53%), followed by the fully fine-tuned VGG16 (71.02%). Explainable AI techniques (Grad-CAM and SWTformer-v1) applied to the given dataset showed that VGG16 focused on broader structural patterns in cell groupings, while SWTformer-v1 attended to more localized regions. These findings suggest that deep-learning models can identify distinguishing image patterns that contribute to classifying RBC images from MS and healthy controls. The XAI analyses further provide insight into the image regions the models use, although their biological relevance requires validation in larger cohorts.

## 1. Introduction

Multiple sclerosis (MS) is a chronic inflammatory and neurodegenerative condition of the central nervous system, causing a broad range of symptoms, including sensory and motor difficulties, coordination problems, and cognitive impairment. It is a leading cause of neurological disability in the young adult population [1]. There has been considerable progress in the relapsing-remitting forms of the condition. Treatment options for progressive forms of MS, however, remain restricted by an incomplete understanding of disease processes [2]. A large body of research has investigated the role of immunological factors in MS [2]. An area of growing research interest in MS is red blood cells (RBCs) [3]. RBCs bind and transport oxygen to tissues and are therefore crucial to cell survival throughout the body. To do this, they contain large amounts of hemoglobin, which is neurotoxic when it escapes from RBCs and enters brain tissue [4]. A number of changes in RBCs in MS have been described, including increased osmotic fragility, enzymatic alterations, and morphological changes [3,4,5,6,7]. Most studies, however, involve relatively small sample sizes because techniques such as osmotic fragility testing and morphological analysis are labor-intensive. This makes it difficult to study a condition like MS conclusively, which is characterized by substantial inter-subject variability in disease course, inflammatory activity, and progression.

In recent years, deep learning has shown considerable promise in medical image classification, achieving strong results in areas such as radiology [8,9], pathology [10], prostate biopsy images [11], and blood cell analysis [12]. Convolutional neural networks (CNNs) are particularly well suited to microscopy tasks because they learn relevant visual features directly from pixel data, removing the need for manual feature engineering [13]. Transformer-based architectures, such as the Swin Transformer, have further expanded the toolbox by introducing attention mechanisms that capture both local detail and broader spatial context within an image [14].

This study does not aim to develop a diagnostic tool for Multiple Sclerosis (MS), but rather to investigate whether blood cell images contain features or patterns associated with MS that can be distinguished from those observed in healthy controls. In this context, deep-learning-based classification serves as an exploratory approach to determine whether blood cells contain information relevant to MS. A successful classification outcome may indicate that disease-associated features are present in blood-cell images and could provide a complementary data modality for further investigation of the disease. Given the limited number of subjects in the present study, the findings should be considered preliminary and are not intended to establish clinical diagnostic performance. Instead, this work provides an initial assessment of the potential of blood cell image analysis to improve understanding of MS and motivates further investigation with larger, more diverse cohorts.

This study examines the performance of four deep learning architectures, VGG16, DenseNet201, MobileNetV2, and the Swin Transformer, in terms of their ability to classify RBC microscopy images into Healthy Control (HC) and MS-affected (MS) categories. The study used 1944 images after applying data augmentation and various preprocessing techniques discussed in Section 3. All four models are evaluated using subject-level Leave-One-Out Cross-Validation (LOOCV) across 13 subjects, ensuring that no subject data appeared in both the training and testing sets. Additionally, explainable AI methods such as Grad-CAM and SWTformer-v1 are used to visualize which image regions influenced each model’s predictions, providing interpretable feedback for medical professionals.

The paper is organized as follows. Section 2 covers related work in blood cell classification and deep learning for medical imaging. Section 3 presents the dataset, preprocessing pipeline, model architectures, and evaluation strategy. Section 4 reports the experimental results. Section 5 presents conclusions and future research directions.

## 2. Related Work

The classification of blood cells using image analysis has been an active area of research, with approaches evolving from traditional image processing to machine learning and, more recently, deep learning methods. Early work in this domain relied on image processing techniques combined with classical machine learning classifiers. Patil and Khot [15] used morphological operations and Sobel edge detection to count and segregate abnormal blood cells, achieving 83% accuracy. Akrimi et al. [16] applied Support Vector Machines (SVMs) to classify RBCs as normal or abnormal based on color, texture, and geometric features extracted from preprocessed images, achieving 99.8% accuracy. Maity et al. [17] proposed a rule-based ensemble algorithm for erythrocyte classification using watershed segmentation, achieving 97.81% accuracy across eight morphological categories. Chy et al. [18] compared Extreme Learning Machine (ELM), SVM, and KNN classifiers for sickle cell detection, with ELM achieving the best performance at 87.73% accuracy.

With the growth of deep learning, CNN-based approaches have become increasingly common for blood cell classification. Alzubaidi et al. [19] proposed an 18-layer CNN framework to classify RBCs into normal, abnormal, and miscellaneous categories, reaching 92.06% accuracy. Xu et al. [20] applied a deep CNN to classify sickle cells, achieving results comparable to those obtained with a deep CNN. Yao et al. [21] introduced a weighted, optimized deformable CNN for white blood cell classification, outperforming classical models such as VGG16, VGG19, and ResNet50. Butt and de Keijzer [12] used transfer learning with VGG16 and ResNet50 to classify Lorrca Ektacytometry microscope images of sickle cells and healthy RBCs, finding that VGG16 achieved 98% test accuracy—a significant improvement over custom CNN architectures that could not exceed 51%.

Transfer learning has proven particularly valuable for small medical imaging datasets. Rather than training from scratch, pre-trained models such as VGG16, ResNet50, and DenseNet can be fine-tuned on domain-specific data, leveraging features learned from large-scale datasets like ImageNet [22,23]. This approach reduces data requirements and training time while often improving classification performance.

Beyond CNNs, researchers have recently applied transformer-based architectures to blood cell and medical image classification. The Swin Transformer [14] introduces a hierarchical architecture with shifted-window self-attention, enabling it to capture both local and global image features efficiently. Chen et al. [24] applied a Shifted Window Vision Transformer to blood cell classification, achieving competitive performance but noting that the resulting attention maps did not always align with established medical knowledge, underscoring the importance of model interpretability.

Explainable AI (XAI) has become essential in healthcare, as clinical adoption requires transparency in model decisions. Grad-CAM [25] is one of the most widely used XAI techniques for CNNs, generating heatmaps that highlight which image regions contribute most to a prediction. For transformer-based models, methods such as SWTformer-v1 [26] provide analogous visualizations by leveraging the final patch tokens and classifier weights to produce class activation maps.

The study compares CNN and transformer architectures (VGG16, DenseNet201, MobileNetV2, and Swin Transformer) on the same dataset, using subject-level LOOCV because the clinical dataset is small. Finally, it applies XAI techniques to both model families, offering interpretable insights into how CNNs and transformers process RBC microscopy images differently.

## 3. Materials and Methods

### 3.1. Dataset

The dataset used in this study consisted of blood film images captured with a high-resolution microscope (4032 × 1960 pixels) by RR Mechatronics, a company specializing in laboratory automation and hematology instrumentation [27]. The blood samples came from 13 volunteers recruited to the NOII study at the University of Southampton, with informed consent (research ethics committee and institutional approval numbers 11/SC/0204 and 41084.A1, respectively), including healthy subjects and those with progressive multiple sclerosis.

The initial dataset contained 629 images, split into 220 healthy control (HC) images and 409 MS-affected (MS) images. Upon inspection, several images were found to be unusable due to chemical treatment of the blood samples (e.g., CDNB, CHP, Diamide) or duplication. These were removed to ensure the model uses original (untreated) images for all subjects. The images were organized into subject-specific folders, and a programmatic check was performed to ensure that images were not duplicated or copied across subject folders. The dataset comprises 13 subjects, forming the basis for the subject-level cross-validation strategy described in Section 3.4.

### 3.2. Data Preprocessing

To further increase dataset diversity and improve model generalization, various image processing techniques were applied independently to each original image, producing multiple enhanced versions of each sample. This process of expanding the dataset by applying different transformations is known as data augmentation. For this study, a greyscale version of each original image was produced, i.e., a single-channel intensity representation that preserves cell morphology while removing color-related noise from staining and illumination variation [28]. Images were stored as three-channel RGB to maintain compatibility with pre-trained architectures. To enhance the visibility of structural details, the HSV color space variant of each image is obtained by multiplying the value (V) channel by a factor of 1.6 to maximize edge visibility without introducing noise [28]. To help the model focus on morphological features rather than memorize specific color patterns, Hue and saturation scaling were applied by shifting the hue channel and multiplying the saturation channel by 1.5, thereby increasing color diversity [28].

**Figure 1:**
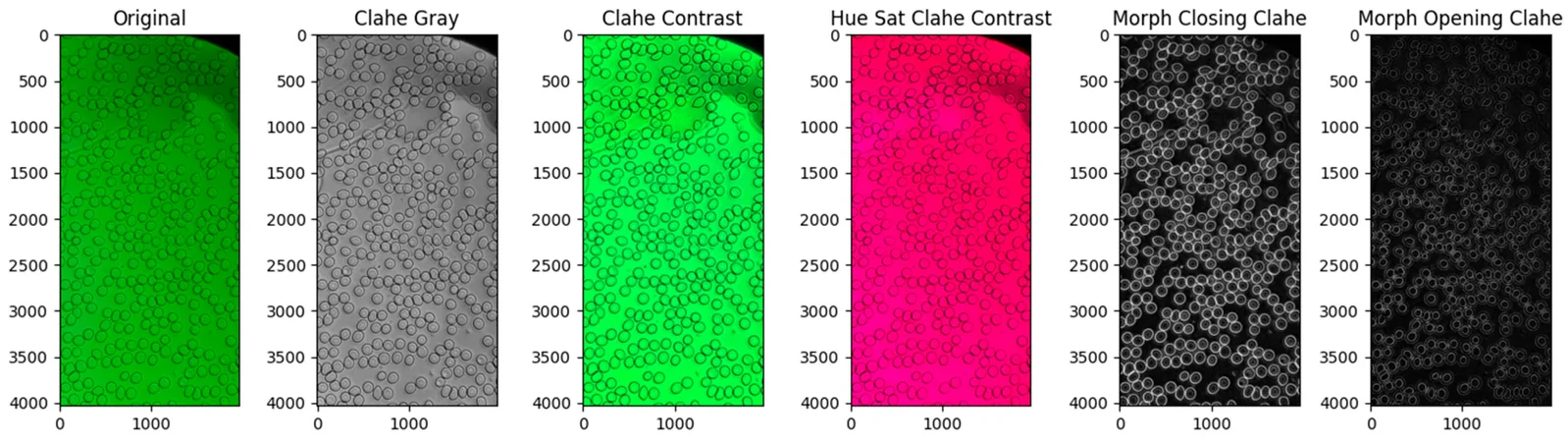
Image preprocessing techniques.

Morphological closing and opening operations are also applied to augment the dataset, e.g., closing estimates and subtracting the image background using a 51 × 51 elliptical kernel to remove shadows and lighting inconsistencies, producing cleaner cell boundaries [29]. Opening removes small bright artifacts using a 15 × 15 elliptical kernel, complementing the closing operation to normalize lighting variation across the dataset [29]. Furthermore, to enhance local contrast and sharpen subtle morphological details, CLAHE (Contrast Limited Adaptive Histogram Equalization) was applied using a tile size of 8 × 8 pixels and a clip limit of 2 [30]. Figure 1 shows a screenshot of multiple variants of a single image.

To preserve subject-level independence and prevent data leakage, the images were organized into separate folders for each subject, and data augmentation was performed independently within each subject folder using a Python (3.11.0) based augmentation procedure. The augmented images remained associated with their respective original subjects. In contrast, the held-out subject folder was kept completely separate during each LOSO iteration and was not used for model training.

This step of data augmentation expanded the dataset to 1944 images (984 HC, 960 MS), including both original and enhanced representations of each sample. The second preprocessing step was image resizing and normalization. The original images were resized to evaluate the effect of input resolution on model performance. Resizing was performed using Lanczos interpolation, a high-quality resampling method that preserves edges and fine details [31]. The 560 × 272 resolution was selected to capture maximum detail while maintaining computational efficiency (see Section 4). All images were normalized using the ImageNet mean ([0.485, 0.456, 0.406]) and standard deviation ([0.229, 0.224, 0.225]) to align the input distributions with the pre-trained model weights.

### 3.3. Models

Four deep learning architectures were selected for evaluation based on their demonstrated effectiveness in medical image [11] and blood cell classification tasks [12,19,25]. The four models were selected to provide a diverse comparison of established and modern deep-learning architectures with different approaches to feature extraction. VGG16 was included as a well-established CNN architecture, providing a relatively straightforward baseline for image classification. DenseNet201 was selected because its dense connectivity promotes feature reuse and enables information to propagate across multiple network layers, which can be beneficial for identifying subtle image features. MobileNetV2 was included as a lightweight and computationally efficient architecture, allowing us to assess whether competitive classification performance can be achieved with a substantially more compact model. Finally, Swin Transformer was selected as a transformer-based architecture to investigate the potential of self-attention and hierarchical feature representation for capturing distinguishing patterns that may differ from those learned by conventional CNNs. Together, these four architectures enable a comparison of model complexity and feature-learning strategies, allowing us to determine which architecture is most suitable for classifying blood-cell images.

#### 3.3.1. VGG16

**Figure 2:**
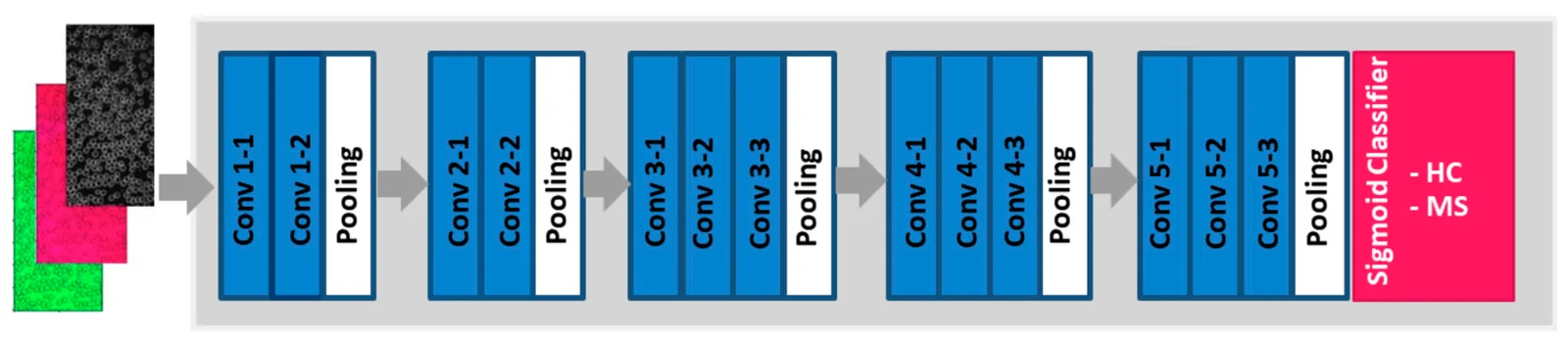
Architecture of the VGG16 model.

VGG16 is a deep convolutional neural network introduced by Simonyan and Zisserman [22] from the Visual Geometry Group at the University of Oxford. The architecture, as shown in Figure 2, consists of 16 learnable layers: 13 convolutional layers organized into five blocks, followed by 3 fully connected layers. A defining characteristic of VGG16 is its exclusive use of small 3 × 3 convolutional filters throughout the network. While individually these filters capture only small local patterns, stacking multiple 3 × 3 layers achieves the same effective receptive field as larger filters (e.g., two 3 × 3 layers cover the same area as one 5 × 5 filter) while using fewer parameters and introducing additional non-linearity through the activation functions between layers.

The five convolutional blocks progressively increase the number of filters: 64 in the first, 128 in the second, 256 in the third, and 512 in both the fourth and fifth blocks. Each block is followed by a max-pooling layer with a 2 × 2 window, which halves the spatial dimensions at each stage. This design creates a characteristic funnel shape where spatial resolution decreases while feature depth increases, transitioning from fine-grained local features in the early layers to higher-level abstract representations in the deeper layers.

After the convolutional blocks, the feature maps are flattened and passed through two fully connected layers of 4096 neurons each, followed by the output layer, as shown in Figure 2. VGG16 was trained under three training settings to examine how the degree of adaptation to the RBC dataset affects classification performance, and these are:

Feature extraction (FE): All convolutional layers were frozen with pre-trained ImageNet weights, and only the classifier head was trained. This tests whether generic image features are sufficient for RBC classification.

Partial fine-tuning (PFT): The final convolutional block (block 5) was unfrozen along with the classifier head, allowing the network to adapt its highest-level feature representations to the dataset while retaining general low-level features from ImageNet.

Full fine-tuning (FFT): All network layers were made trainable, giving the model maximum flexibility to learn dataset-specific features at every level of the hierarchy.

#### 3.3.2. DenseNet201

**Figure 3:**
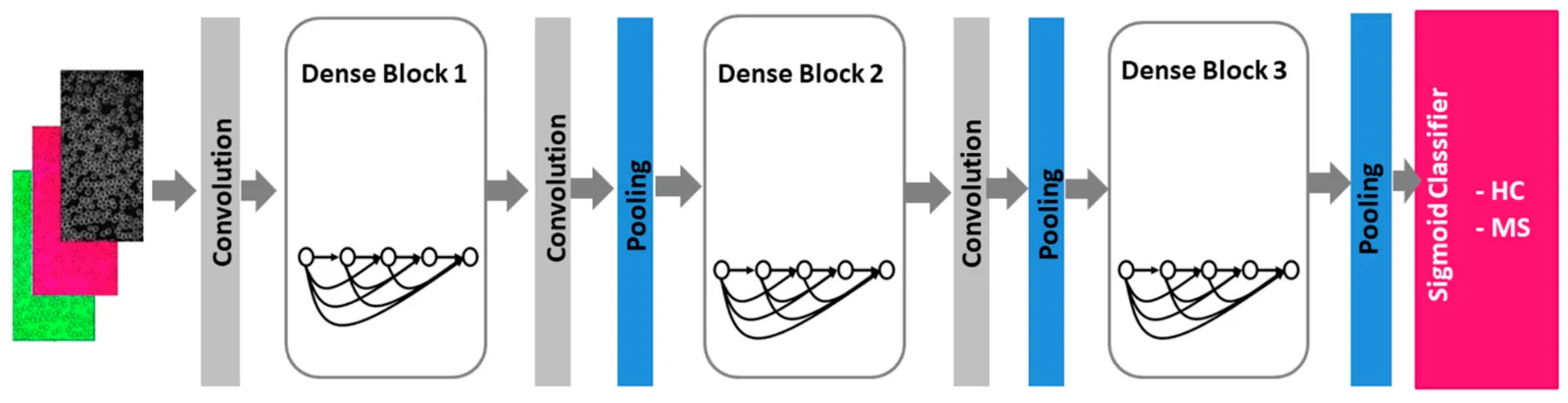
Architecture of DenseNet.

DenseNet201 (Densely Connected Convolutional Network), as shown in Figure 3, introduces a fundamentally different connectivity pattern from sequential architectures such as VGG16 [32]. Within each dense block, every layer receives the feature maps of all preceding layers as input and passes its own feature maps to all subsequent layers. This is achieved through concatenation rather than summation, meaning feature maps accumulate as they pass through the block. This dense connectivity encourages feature reuse across the network and helps maintain strong gradient flow during training, reducing the risk of vanishing gradients in very deep networks [29].

The DenseNet201 architecture consists of four dense blocks, each separated by a transition layer. Within each dense block, feature map spatial dimensions remain constant to allow direct concatenation. The transition layers between blocks serve two purposes: a 1 × 1 convolution compresses the accumulated channel depth, and an average pooling operation halves the spatial dimensions to maintain computational efficiency.

Despite having 201 layers, the dense connectivity and channel compression through transition layers keep the parameter count manageable. For this study, the original classifier head was replaced with a 2-output head for binary classification. DenseNet201 was evaluated solely for feature extraction.

#### 3.3.3. MobileNetV2

MobileNetV2 is designed for computational efficiency, making it suitable for resource-constrained environments without sacrificing classification performance [33]. The key innovation is the use of depthwise separable convolutions, which decompose a standard convolution into two separate operations: a depthwise convolution that applies a single filter per input channel (capturing spatial patterns), followed by a 1 × 1 pointwise convolution that combines information across channels. This factorization dramatically reduces the number of computations and parameters compared to standard convolutions.

**Figure 4:**
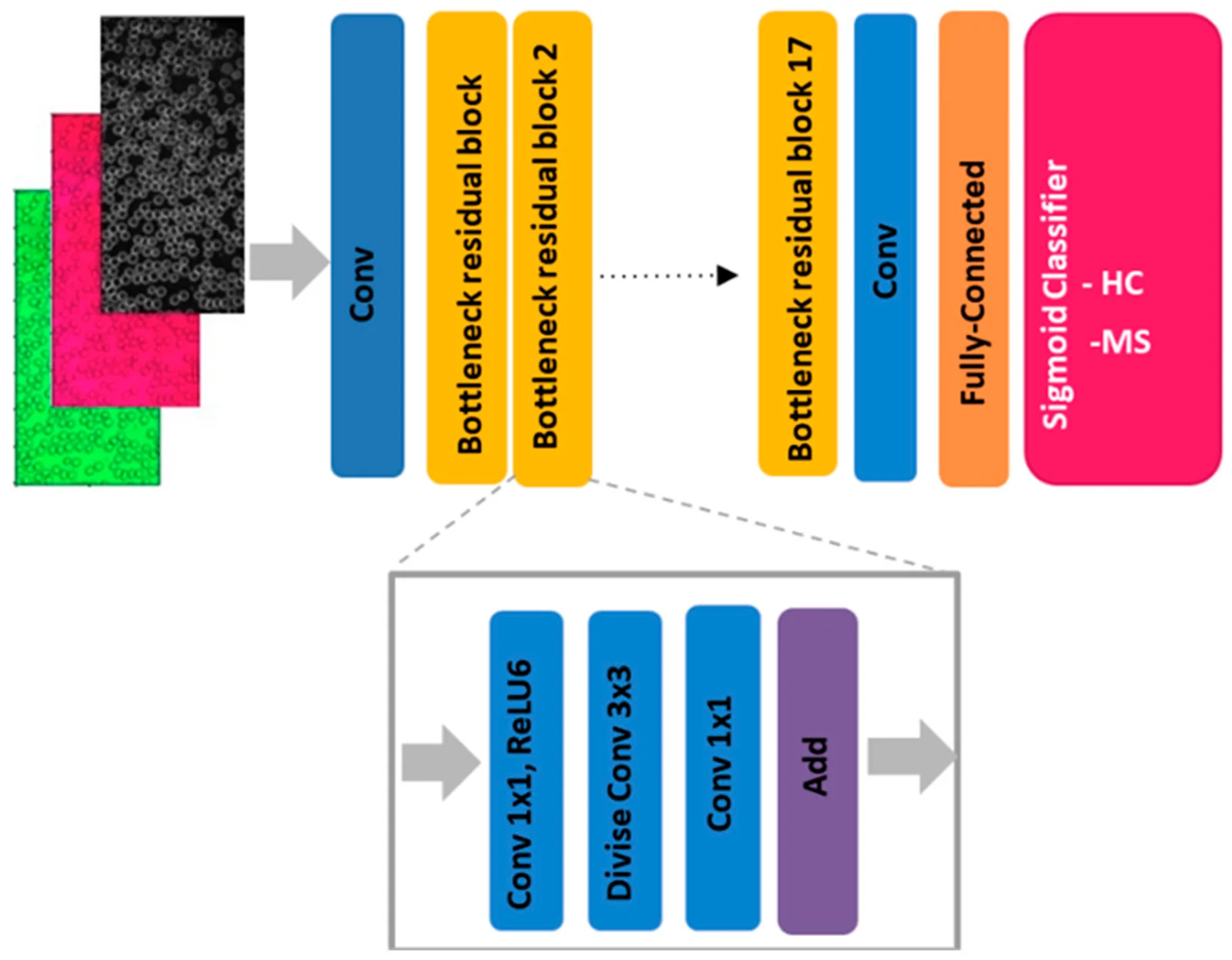
Architecture of MobileNetV2.

The architecture, as shown in Figure 4, is built around inverted residual blocks, which follow an expansion-projection pattern. Each block first expands the input to a higher-dimensional feature space using a 1 × 1 convolution (allowing the model to learn complex patterns), applies a depthwise convolution for spatial filtering, and then projects back to a compact lower-dimensional representation with another 1 × 1 convolution. Residual connections link the input and output of each block when their dimensions match, enabling efficient gradient flow. The expansion and projection steps use ReLU6 activation, while the final projection uses a linear activation to prevent information loss in the low-dimensional bottleneck.

#### 3.3.4. Swin Transformer

**Figure 5:**
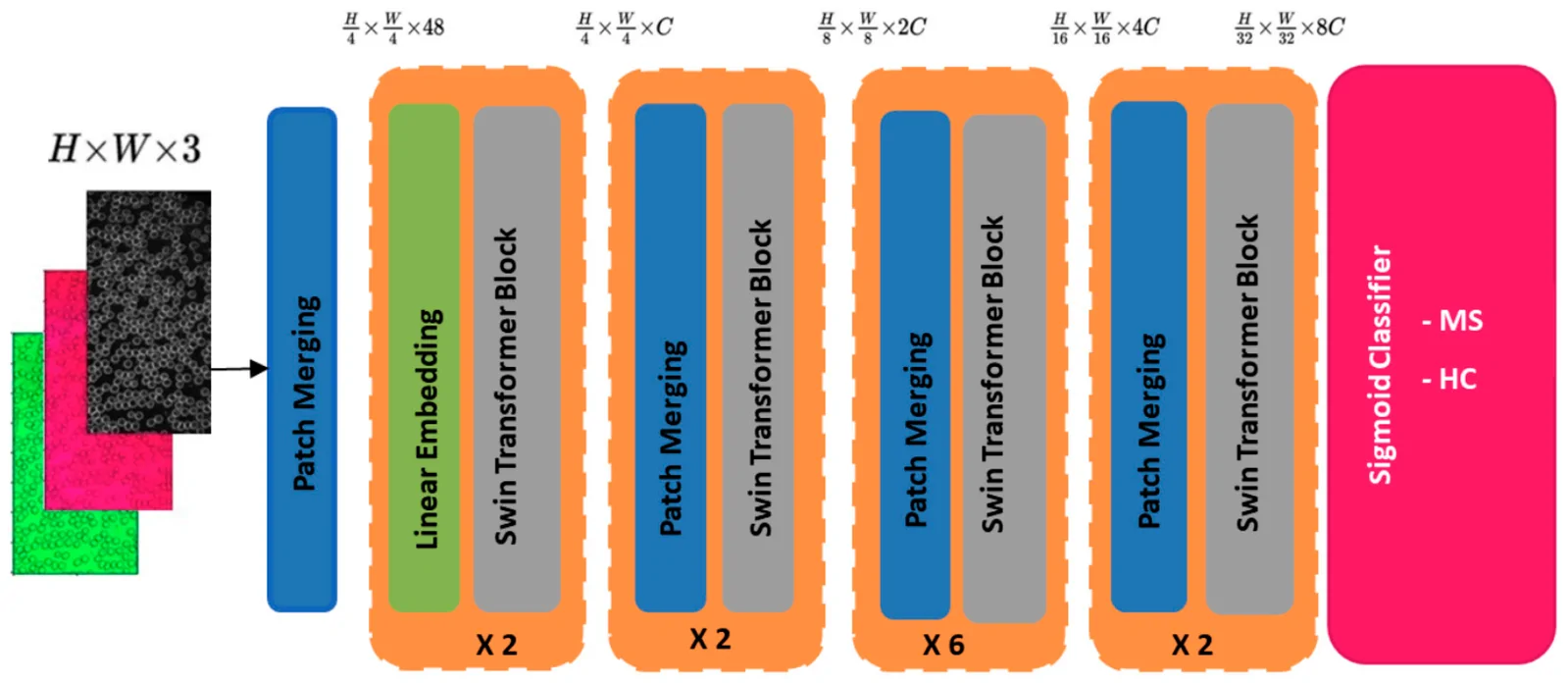
Basic Architecture of Swin Transformer (Tiny).

The Swin Transformer, as shown in Figure 5, departs from CNN-based approaches by using a self-attention mechanism rather than convolutions to model relationships between image regions [14,24]. Unlike standard Vision Transformers, which compute attention across the entire image at once—a computationally expensive step for high-resolution inputs—the Swin Transformer restricts attention to local windows and gradually integrates information across the image through a hierarchical design.

The input image is first divided into non-overlapping 4 × 4-pixel patches, which are flattened and projected into feature vectors via a linear embedding layer. These patch tokens are then processed through four sequential stages. Between stages, patch-merging layers concatenate groups of 2 × 2 neighboring patches and project them into a higher-dimensional vector, reducing the total number of tokens by a factor of 4 while increasing feature depth. This creates a hierarchy in which early stages operate on high-resolution representations that capture fine details, and later stages operate on compact representations that encode broader structure—similar to multi-scale feature extraction in CNNs.

Within each stage, self-attention is computed independently over local 7 × 7 patch windows. Each patch token is projected into query (Q), key (K), and value (V) representations:

Q = XW_Q_, K = XW_K_, V = XW_V_ (1)

Attention is then computed as shown in Equation (2)

(2) $$\mathrm{Attention}(Q,K,V)=\mathrm{softmax}(\frac{QK^{T}}{\surd d})V$$

where d is the feature dimension. This operation determines how strongly each patch should attend to its neighbors within the same window. Multi-head self-attention runs several of these operations in parallel, with each head learning different types of relationships (e.g., fine textures vs. broader shapes). The outputs of all heads are concatenated to produce a rich local feature representation.

To enable information flow between adjacent windows, the Swin Transformer alternates between regular and shifted window partitions across consecutive layers. In shifted layers, the window grid is offset by half the window size, allowing patches at window boundaries to interact with their neighbors in the adjacent window. This mechanism provides cross-window connectivity while preserving the computational efficiency of local attention.

The Swin-T (Tiny) variant is used in this study, configured with 96 initial channels, a block configuration of {2, 2, 6, 2} across four stages, and approximately 28 million parameters (Table 1). Its smaller size relative to the Swin-S, Swin-B, and Swin-L variants makes it suitable for training on limited datasets.

After the final stage, the output feature maps are globally average-pooled into a fixed-length vector and passed through a linear classifier head with 2 output neurons for binary classification.

### 3.4. Setup and Strategy

All four architectures were initialized with weights pre-trained on ImageNet [22]. During fine-tuning, the convolutional or patch embedding layers initially retained their pre-trained weights. At the same time, the classification head was replaced and trained from scratch for the binary HC vs. MS task. For VGG16 under partial and full fine-tuning, deeper layers were progressively unfrozen to adapt the pre-trained features to the microscopy dataset.

For smaller or imbalanced datasets, leave-one-out cross-validation (LOOCV) is often recommended. In LOOCV, *k* = *n*, where *n* is the number of samples in the dataset, meaning that all samples except one are used for training in each iteration. Because the model must be trained *n* times, LOOCV is computationally demanding. As a result, it is generally not suitable for very large datasets or for models with computationally expensive training procedures [34].

A subject-level LOOCV strategy was used to evaluate model performance. In each fold, images from one of the 13 subjects were held out as the test set, while images from the remaining 12 subjects were used for training. This process was repeated for all 13 subjects, ensuring that no subject’s data appeared in both the training and test sets within the same fold. LOOCV is well-suited to small datasets as it maximizes the available training data in each iteration [35]. All models were implemented in PyTorch (2.7.1), with architectures accessed through the timm library. CNN models under feature extraction were trained for 10 epochs, while fine-tuned CNNs and the Swin Transformer were trained for up to 20 epochs. Early stopping with a patience of 5 epochs was applied based on validation accuracy, using 15% of the training data as a validation set. The AdamW optimizer [36] was used with cross-entropy loss [37] for all experiments. The training configurations for the models were selected to ensure reproducibility. All models were trained using a learning rate of 1 × 10^−4^, a batch size of 32, and a random seed of 42. The models were trained using an NVIDIA RTX 3000 Ada Generation Laptop GPU.

## 4. Results

The training process was carried out in three phases. During the first phase, three pretrained CNN models were used to extract features by freezing their convolutional bases. Table 2 summarizes the patient-wise LOOCV accuracies for each model.

Among the three CNN models, VGG16 achieved the highest mean LOOCV accuracy at 60.44%, followed by MobileNetV2 at 53.16% and DenseNet201 at 43.57%. Based on this result, VGG16 was selected for further experiments in the second phase, with both partial and full fine-tuning. The results of that phase are presented in Table 3. Partial fine-tuning improved the mean accuracy to 65.62%, while full fine-tuning further increased it to 71.02%. This indicates that allowing the model to adapt its learned features to the RBC dataset yields better classification performance, albeit at the cost of increased training time.

In the third phase, the Swin Transformer was evaluated at three input resolutions to investigate how input image resolution affects transformer-based feature extraction. Three input resolutions (224 × 224, 560 × 272, and 1024 × 512) were evaluated specifically with the Swin Transformer. These resolutions were selected to represent different levels of spatial detail, ranging from the commonly used 224 × 224 input size to higher-resolution representations that better preserve the spatial characteristics of the blood-cell images. The 560 × 272 resolution was selected as an intermediate resolution that preserves the original image aspect ratio while balancing spatial detail and computational requirements, whereas 1024 × 512 was used to investigate whether substantially higher spatial resolution could yield additional discriminative information. Table 4 summarizes the results. The 560 × 272 resolution achieved the highest mean accuracy of 73.53%, outperforming both the 224 × 224 (66.14%) and 1024 × 512 (59.60%) configurations. The smaller resolution likely discarded too much morphological detail, while the larger resolution may have introduced excessive complexity that hindered learning.

Table 5 presents a side-by-side comparison of the best-performing configurations: the Swin Transformer (560 × 272) and fully fine-tuned VGG16. Both were trained using the same input resolution and evaluation setup.

The Swin Transformer slightly outperformed VGG16 in mean accuracy (73.53% vs. 71.02%). Both models achieved strong results for subjects such as MS_J36, MS_J38, and MS_J40, but struggled with MS_J42. The performance gap between the two models varied considerably across individual subjects.

A paired t-test was conducted to determine whether the difference in subject-wise accuracies between the Swin Transformer and VGG16 (FFT) was statistically significant. The resulting *p*-value was 0.66, well above the significance threshold of α = 0.05. This indicates that the two models perform comparably on this dataset, with no statistically meaningful advantage for either architecture.

## 5. Discussion

The results provide preliminary evidence that deep-learning models can identify distinguishing patterns in red blood cell microscopy images from healthy controls and subjects with MS. The Swin Transformer achieved the highest mean LOOCV accuracy of 73.53%, marginally outperforming the fully fine-tuned VGG16 at 71.02%. The progression from feature extraction to full fine-tuning in VGG16 (60.44% → 65.62% → 71.02%) suggests that the pre-trained ImageNet features, while useful, are not fully sufficient for this task. Allowing deeper layers to adapt to the specific characteristics of RBC microscopy images yielded meaningful gains. This is consistent with broader findings in medical imaging, where domain-specific fine-tuning typically outperforms fixed feature extraction [10,23].

Among the CNN models under feature extraction, DenseNet201 performed the weakest (43.57%), despite its architectural advantages of dense connectivity and feature reuse. This may be attributed to its larger parameter count relative to the small dataset size, making it more prone to overfitting even with a frozen backbone. MobileNetV2 performed moderately (53.16%), suggesting that its lightweight design, while efficient, may lack the capacity to capture the subtle morphological differences relevant to this task.

**Figure 6:**
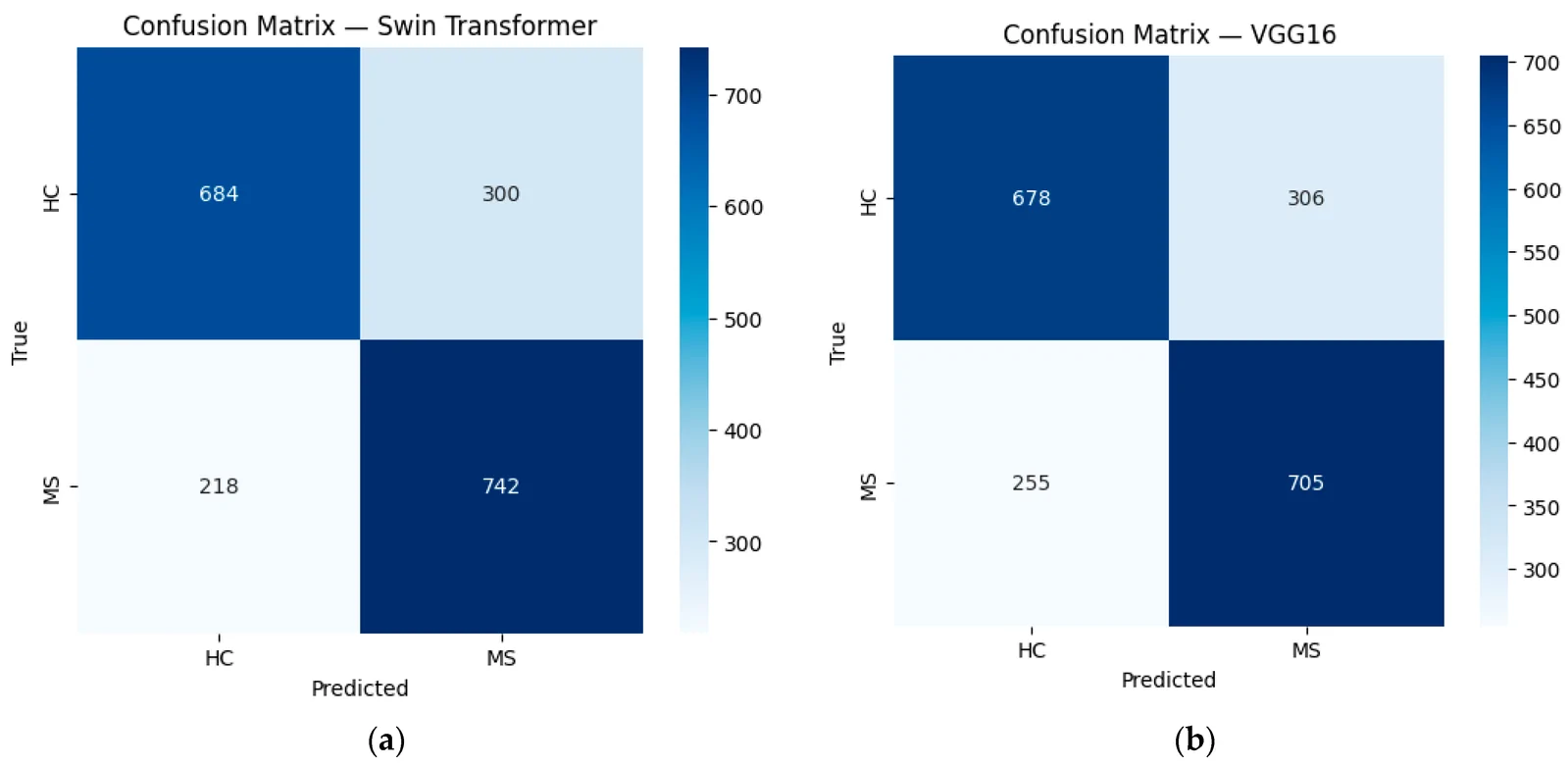
Confusion Matrices: (**a**) Confusion matrix for Swin Transformer. (**b**) Confusion matrix for VGG16.

Figure 6 shows the confusion matrices for the two top-performing models, indicating that both VGG16 and Swin Transformer performed well in differentiating Multiple Sclerosis (MS) samples from healthy controls (HC). This indicates that the models were able to identify image features that contributed to distinguishing the MS and HC classes in this dataset. Nonetheless, the Swin Transformer consistently surpassed VGG16 in most evaluation metrics. From the confusion matrices, the Swin Transformer correctly identified 684 HC samples and 742 MS samples, while VGG16 correctly identified 678 HC and 705 MS samples. Importantly, the Swin Transformer had fewer false negatives for MS (218 vs. 255), reflecting higher sensitivity to MS. Although healthy control accuracy differed little, the Swin Transformer showed more balanced performance across both classes.

The classification reports for both models, as shown in Table 6, demonstrate that the Swin Transformer outperformed VGG16 across all major classification metrics. For the MS class, Swin Transformer achieved higher precision (71.21% vs. 69.73%), sensitivity (77.29% vs. 73.44%), and F1-score (74.13% vs. 71.54%). The higher sensitivity is particularly relevant for MS classification, as it indicates that the Swin Transformer correctly identified a greater proportion of MS samples. Similarly, the macro-averaged F1-score was higher for Swin Transformer (73.33%) than for VGG16 (71.14%), indicating a modest but consistent improvement across both classes. Overall, these results suggest that the Swin Transformer’s attention-based architecture offers a slight advantage over the convolutional VGG16 architecture for capturing discriminative patterns in blood cell images.

**Figure 7:**
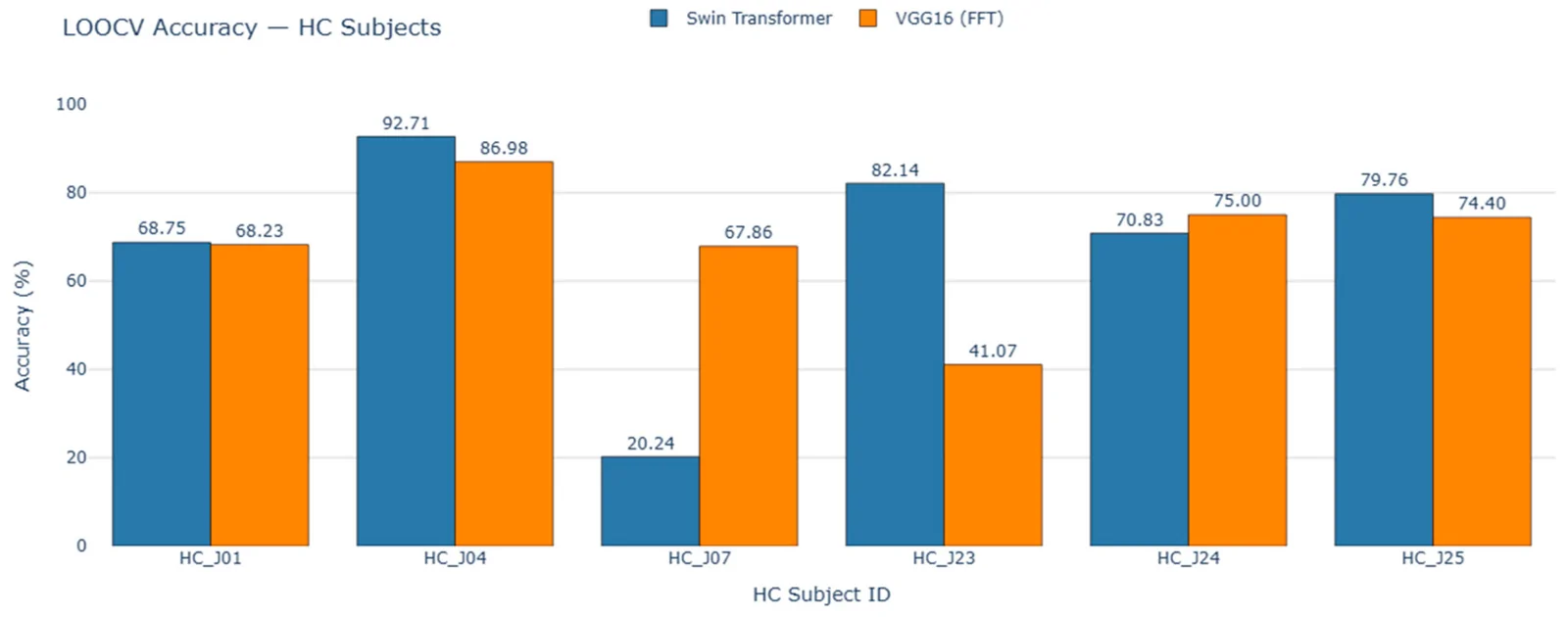
Subject-wise accuracy for HC subjects comparing Swin Transformer and VGG16.

**Figure 8:**
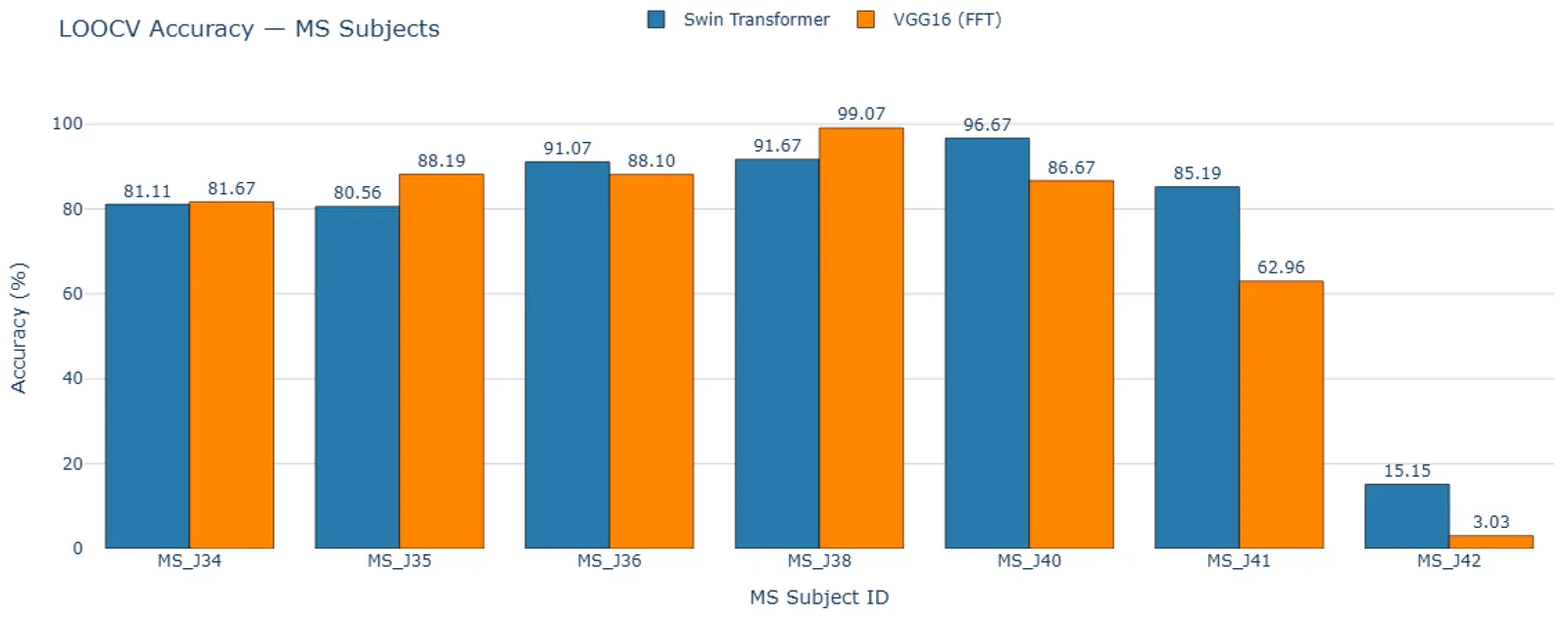
Subject-wise accuracy for MS subjects comparing Swin Transformer and VGG16.

Subject-wise analysis reveals substantial variability across subjects, as shown in Figure 7 and Figure 8. While both models achieve high accuracy for certain subjects (e.g., MS_J38 in Figure 8), performance drops significantly for others (e.g., MS_J42 in Figure 8). These results suggest that inter-subject variability in red blood cells has a stronger impact on classification performance than the choice of model architecture.

The comparable performance of both models can be attributed to their distinct feature-extraction mechanisms. VGG16, a convolutional neural network pretrained on ImageNet, relies on hierarchical convolutional filters to extract local texture and shape information.

**Figure 9:**
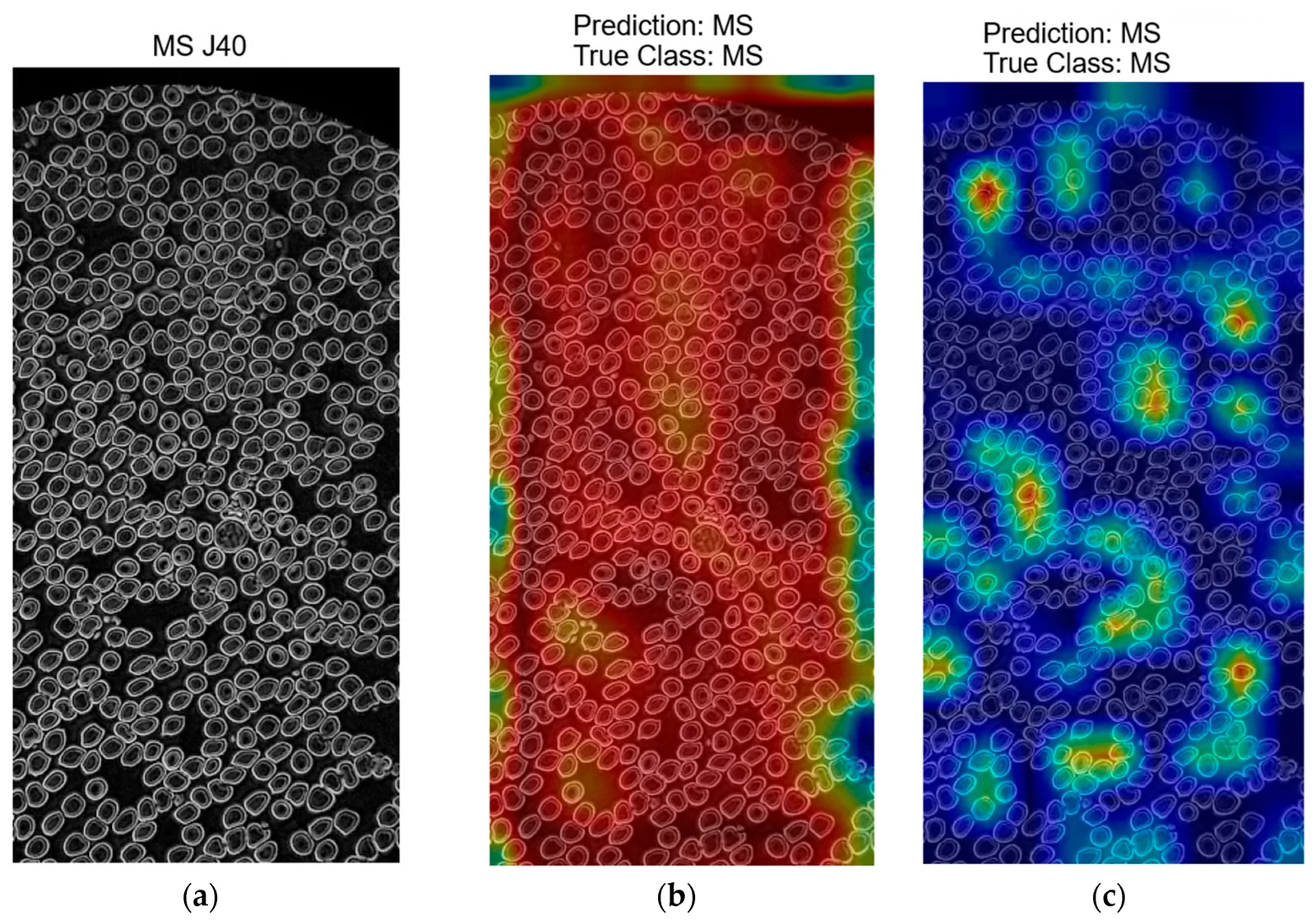
Heatmaps: (**a**) Test Image1. (**b**) Grad-CAM. (**c**) SWTformer-v1.

**Figure 10:**
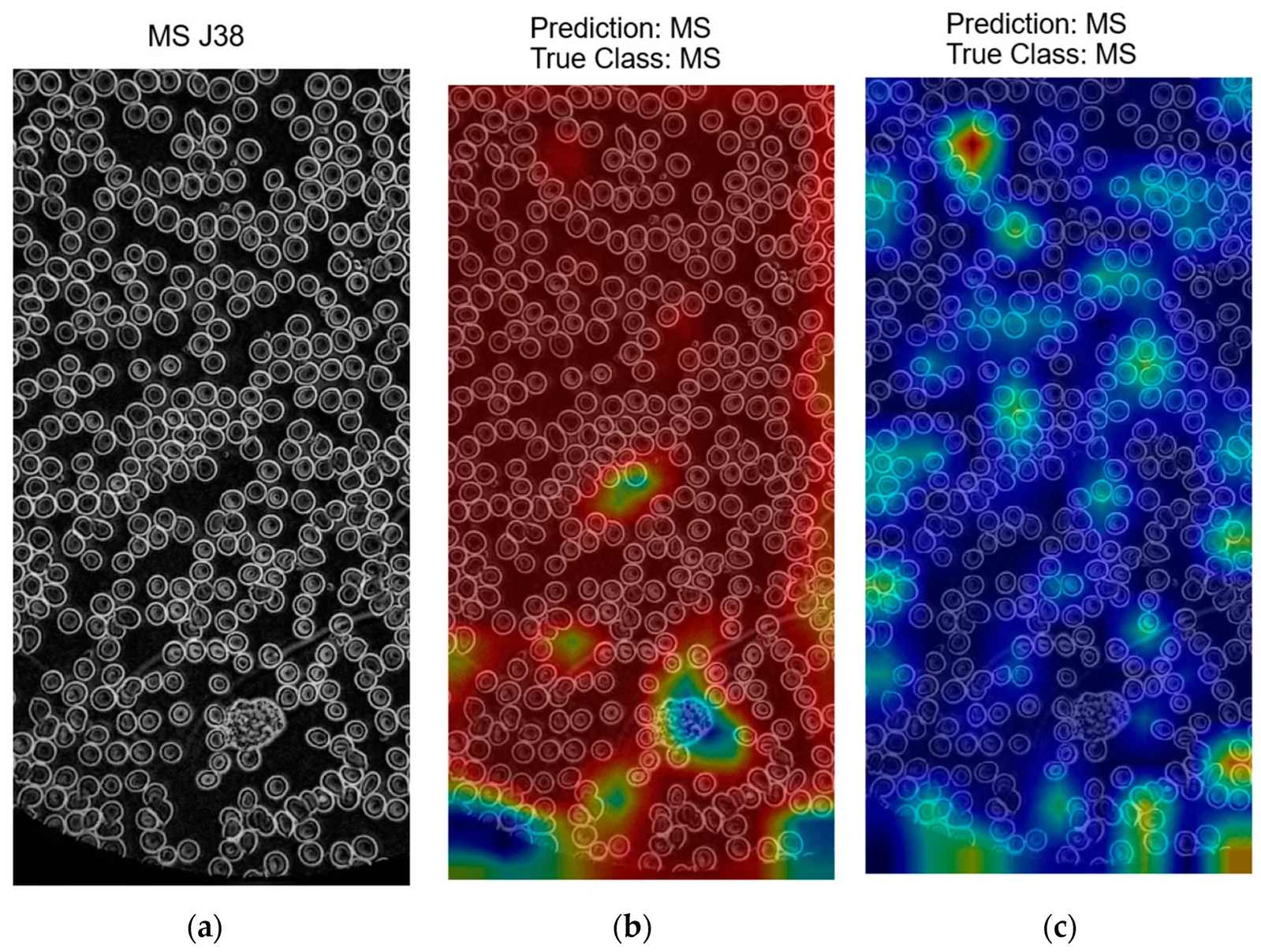
Heatmaps: (**a**) Test Image2. (**b**) Grad-CAM. (**c**) SWTformer-v1.

**Figure 11:**
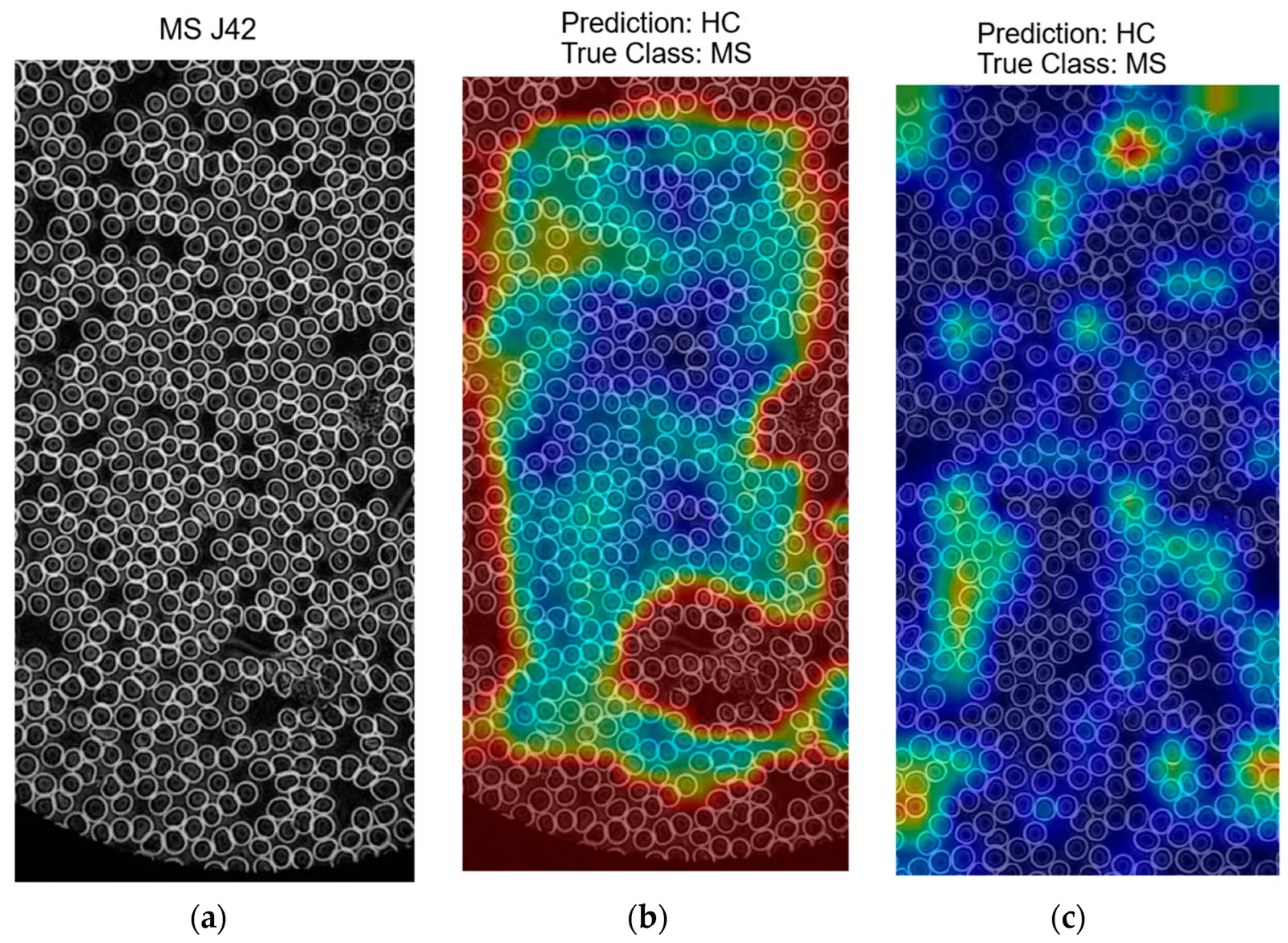
Heatmaps: (**a**) Test Image. 3 (**b**) Grad-CAM. (**c**) SWTformer-v1.

To interpret the models’ performance, Grad-CAM and SWTformer-v1 were used. When fine-tuned on red blood cell images, Grad-CAM visualizations show relatively broad and spatially coherent activation regions, suggesting that the model relies on larger structural patterns for classification. The three test images from the MS class were chosen, with two having true classification and one having false classification. Figure 9 and Figure 10 show that VGG16 highlights broad and spatially coherent regions of attention. In contrast, the Swin Transformer employs a shifted-window self-attention mechanism to model both local and global dependencies. The corresponding SWTformer-v1 visualizations in the study highlight more localized and fragmented regions of attention. While this indicates that the model focuses on specific image regions, the resulting heatmaps are less spatially consistent and do not always align with established medical knowledge [24]. In the misclassified image shown in Figure 11, Grad-CAM highlighted a broader outer region, whereas SWTformer-v1 focused on one or two small, localized regions, indicating different feature patterns that contributed to the incorrect HC prediction.

This suggests that although the Swin Transformer is designed to capture broader contextual relationships, its explainability in this application is less intuitive than that of CNN-based approaches. Therefore, interpretability methods for transformer-based architectures may require further refinement. These visualizations should not be interpreted as evidence that the highlighted regions or cell clustering represent confirmed MS-related biological features. Rather, they provide an interpretable view of the patterns the models use for classification. Establishing whether these patterns have a genuine biological association with MS will require further investigation using larger cohorts and complementary biological and clinical data.

The findings are consistent with recent studies showing that transformer-based architectures perform competitively in both medical and non-medical image analysis tasks. In non-medical domains such as deepfake detection, transformer-based models have been shown to match or outperform CNN-based architectures in capturing subtle visual inconsistencies [38].

In medical imaging applications such as glaucoma classification, comparative results between VGG16 and Swin Transformer (among others) show that VGG16 achieved the highest overall accuracy. In contrast, Swin Transformer achieved higher recall for the positive class, indicating a trade-off between sensitivity and overall accuracy rather than clear performance dominance [39]. In blood cell classification tasks, shifted-window transformer architectures have been shown to outperform CNN-based architectures and standard Vision Transformers [24].

Shifted-window transformer architectures have shown performance comparable to convolutional neural networks such as VGG16, although their potential advantages in capturing image features have not consistently resulted in statistically significant improvements in classification accuracy. An important limitation of this study is the relatively small number of independent subjects (13), despite the larger number of images obtained through data augmentation. The augmentation procedures generated additional image variations within each subject folder but did not increase the number of independent biological samples or the diversity of subjects. To minimize the risk of data leakage, subject-specific data were kept separate throughout the analysis, and a Leave-One-Subject-Out (LOSO) strategy was used, with all images from one subject excluded from model training and reserved for validation while the remaining subjects were used for training. Nevertheless, the limited number of independent subjects may result in variability in model performance and limit the generalizability of the findings. Therefore, the current results should be considered preliminary and further validated in larger, more diverse cohorts.

## 6. Conclusions

This study investigated whether deep learning models can detect signs of multiple sclerosis from red blood cell microscopy images. Four architectures, VGG16, DenseNet201, MobileNetV2, and the Swin Transformer, were evaluated using subject-level Leave-One-Out Cross-Validation on a dataset of 1944 images derived from 13 subjects.

The Swin Transformer achieved the highest mean accuracy of 73.53%, followed closely by the fully fine-tuned VGG16 at 71.02%, with no statistically significant difference between them (*p* = 0.66). The CNN feature extraction experiments identified VGG16 as the strongest performer among the three CNN architectures, and fine-tuning progressively improved its accuracy.

Using a deep learning approach to analyze blood images, a difference in RBC morphology between MS and healthy controls was identified. While these RBC changes are most likely secondary to the MS disease process and may also occur in other chronic diseases, further research is needed to determine whether they may play a role in progression via a positive feedback cycle, rather than merely being an epiphenomenon.

The observed inter-subject variability highlights the challenges associated with developing universally generalizable classifiers from image data alone. Future studies should explore multimodal frameworks that combine morphological features extracted from blood cell images with subject-specific metadata and clinical variables. Such approaches may capture complementary disease-related information and reduce performance variability across subjects. Future work should include longitudinal data and a larger, more diverse cohort; confidence intervals and other uncertainty estimates; and independent external validation before the approach can be considered for clinical application.

## Figures and Tables

**Table 1:** Swin Transformer variants and configurations.

Model	Initial Channels (C)	Block Configuration	Parameters (M)
Swin-T (Tiny)	96	{2, 2, 6, 2}	28 M
Swin-S (Small)	96	{2, 2, 18, 2}	50 M
Swin-B (Base)	128	{2, 2, 18, 2}	88 M
Swin-L (Large)	192	{2, 2, 18, 2}	197 M

**Table 2:** LOOCV performance of pretrained models for feature extraction.

Subject	DenseNet201 (FE)	MobileNetV2 (FE)	VGG16 (FE)
HC_J01	16.15	40.62	51.04
HC_J04	3.65	6.25	34.9
HC_J07	36.31	52.38	53.57
HC_J23	22.02	56.55	65.48
HC_J24	80.21	97.92	71.88
HC_J25	61.9	73.81	76.19
MS_J34	81.67	92.78	84.44
MS_J35	72.22	81.25	96.53
MS_J36	58.93	45.24	58.93
MS_J38	75	76.85	94.44
MS_J40	20.83	26.67	43.33
MS_J41	35.19	32.41	36.11
MS_J42	2.27	8.33	18.94
Mean LOOCV Acc.	43.57	53.16	60.44

**Table 3:** LOOCV accuracies for VGG16 under three training configurations.

Subject	VGG16 (FE)	VGG16 (PFT)	VGG16 (FFT)
HC_J01	51.04	70.83	68.23
HC_J04	34.90	90.10	86.98
HC_J07	53.57	47.02	67.86
HC_J23	65.48	42.26	41.07
HC_J24	71.88	46.88	75
HC_J25	76.19	55.95	74.40
MS_J34	84.44	68.89	81.67
MS_J35	96.53	98.61	88.19
MS_J36	58.93	89.88	88.10
MS_J38	94.44	87.04	99.07
MS_J40	43.33	58.33	86.67
MS_J41	36.11	80.56	62.96
MS_J42	18.94	16.67	3.03
Mean LOOCV Acc.	60.44	65.62	71.02

**Table 4:** Swin Transformer LOOCV accuracies across input resolutions.

Subject	Acc 224 × 224 (%)	Acc 1024 × 512 (%)	Acc 560 × 272 (%)
HC_J01	69.79	39.06	68.75
HC_J04	84.9	9.38	92.71
HC_J07	26.79	41.67	20.24
HC_J23	72.02	46.43	82.14
HC_J24	56.25	85.42	70.83
HC_J25	66.67	82.14	79.76
MS_J34	83.89	97.78	81.11
MS_J35	11.11	100	80.56
MS_J36	95.83	84.52	91.07
MS_J38	90.74	80.56	91.67
MS_J40	80	51.67	96.67
MS_J41	62.04	50.93	85.19
MS_J42	59.85	5.3	15.15
Mean Accuracy	66.14	59.60	73.53

**Table 5:** Subject-wise LOOCV accuracies (%) for Swin Transformer and VGG16 (FFT).

Subject	Swin Transformer	VGG16 (FFT)
HC_J01	68.75	68.23
HC_J04	92.71	86.98
HC_J07	20.24	67.86
HC_J23	82.14	41.07
HC_J24	70.83	75
HC_J25	79.76	74.40
MS_J34	81.11	81.67
MS_J35	80.56	88.19
MS_J36	91.07	88.10
MS_J38	91.67	99.07
MS_J40	96.67	86.67
MS_J41	85.19	62.96
MS_J42	15.15	3.03
Mean LOOCV Acc.	73.53	71.02

**Table 6:** Classification report of MS subjects.

Model	Precision	Recall/Sensitivity	F1-Score	Specificity	Macro-Average (F1-Score)
Swin Transformers	71.21%	77.29%	74.13%	69.51%	73.33%
VGG16(FFT)	69.73%	73.44%	71.54%	68.90%	71.14%

## Data Availability

The dataset used in this study is not publicly available or publicly archived because it is limited in size and contains a small number of independent subjects. Public release of the dataset is therefore not currently planned.
